# Equivalent Circuit Modeling and Analysis for Microfluidic Electrical Impedance Monitoring of Single-Cell Growth

**DOI:** 10.3390/bios15020113

**Published:** 2025-02-14

**Authors:** Yingying Wang, Haoran Wu, Yulu Geng, Zhao Zhang, Jiaming Fu, Jia Ouyang, Zhen Zhu

**Affiliations:** 1 School of Integrated Circuits, Southeast University, Wuxi Campus, Zhuangyuan Road 5, Wuxi 214000, China; yy_wang1994@seu.edu.cn (Y.W.);; 2 College of Chemical Engineering, Nanjing Forestry University, Longpan Road 159, Nanjing 210037, China

**Keywords:** electrical impedance spectroscopy, equivalent circuit model, microelectrode array, *Saccharomyces cerevisiae*

## Abstract

Microfluidics has significantly advanced the field of single-cell analysis, particularly in studies related to cell growth, division, and heterogeneity. Electrical impedance spectroscopy (EIS), a label-free and non-invasive biosensing technique, has been integrated into microfluidic devices for high-throughput and long-term monitoring of single budding yeast cells. Accurate interpretation of EIS measurements of cell growth dynamics necessitates the establishment of theoretical equivalent circuit models for the single-cell sensing system. Here, we report on the development of equivalent circuit models of an in situ EIS sensing system to elucidate cell growth. Firstly, finite element modeling and simulation of an EIS measurement of cell growth in the EIS sensing unit were performed, guiding the fittings of electrical components for an established equivalent circuit model (ECM). From the ECM, we extracted an equivalent volume fraction applicable to various cell and sensing unit geometries to describe the geometry-dependent sensing characteristics corresponding to the electrical response in the model. Then, EIS measurements of an immobilized cell in a microfluidic device were conducted via peripheral circuits. A lumped parameter model for the entire EIS measurement system was established, with electrical components determined by fitting to experimental data. The rationality of the proposed theoretical model was validated through the long-term impedance variation induced by cell growth in experiments, demonstrating its feasibility in linking EIS data with the bio-physics underlying the experimental phenomenon.

## 1. Introduction

Recent advancements in single-cell analysis have revolutionized the understanding of cellular behaviors, with deeper explorations in cell heterogeneity and regulation pathways. As a fundamental biological process, cell growth and division at single-cell resolution are essential for insights into cell aging, drug development, and cell response upon external stimuli [1,2,3]. The budding yeast, *Saccharomyces cerevisiae*, has served as one of the most widely used eukaryotic model organisms in single-cell analysis [4] for the merits of rapid reproduction, simple cultivation, short life cycle, and ease of genetic manipulation [5,6].

In virtue of rapidly developing microfluidic technologies, accurate methods to study individual yeast cells have emerged, such as single-cell isolation and immobilization [1,2], simultaneous culturing [7], and morphological investigation [8]. Microscopic imaging is a prevalent technique for single-cell analysis based on microfluidic devices, which are capable of high-throughput cell assays [9]. However, microfluidic single-cell analysis usually requires the processing of a large number of cell images, and thus it is highly time-consuming and computationally expensive [10].

By integrating microelectrodes into microfluidic devices, label-free and non-invasive electrical impedance spectroscopy (EIS) has been introduced into single-cell analysis to extract multiple biological parameters. Paired electrodes, such as coplanar electrodes [11] and facing electrodes [12], can be incorporated into an impedance flow cytometer (IFC), which enables single cells sequentially passing through the sensing region [13]. The IFC has been mainly utilized in the instantaneous identification or discrimination of targeted samples from a homogenized suspension [14]. As a part of the electrical sensing system, suspended cells can be conceptualized as an equivalent circuit model (ECM). Based on the simplified dielectric properties of a biosensing system initially proposed in 1989 [15], the ECM has been merged into single-cell analysis simply by adding the circuit components of a resistor that represents the cytoplasm in series with two capacitors that describe the cell membrane [16,17,18]. However, as the sample position was not fixed when passing through the sensing region, a construction channel [19] or sheath-flow focusing [12] were usually employed to improve the precision of sample position when conducting impedance measurements. As a result, cells were mechanically constrained to a specific shape, position, or orientation within the sensing region, affecting the obtained electrical properties and parameters from the impedance data. Moreover, the utilization of transient impedance measurements at a few discrete frequencies limited the capability of interrogating the various dynamic processes of cells comprehensively.

In order to further investigate multiple cell properties across a broad band of frequencies and over an extended time period, electrode pairs can be incorporated with single-cell-trapping structures to perform in situ wide-band electrical impedance measurements of single immobilized cells [20,21]. In previous studies, we developed a series of microfluidic devices that integrated coplanar microelectrodes aligning with bottleneck-like single-cell traps to facilitate the EIS detection of cell growth and daughter-cell dissection of yeast cells [7,22]. Finite element modeling of geometry-dependent sensing characteristics has proved that impedance signals are highly sensitive to cell sizes and the design of the sensing unit. Therefore, wide-band electrical impedance variations could be used to characterize the morphological dynamics induced by cell growth. However, the electrical response of an immobilized cell in an equivalent circuit model with regard to the variations in cell morphology during its growth is still missing. A theoretical model of electrical impedance variations in the characterization of long-term cell growth remains unclear, bringing challenges in the interpretation of time-lapse wide-band EIS signals.

Here, we presented the equivalent circuit models of an in situ EIS sensing system to model the variations in the equivalent electrical parameters induced by cell growth. Initially, an ECM of the EIS sensing unit occupied with a single budding yeast cell was established to theoretically mimic the response of electrical impedance signals upon cell size variation. Electrical components in the ECM were derived by fitting to the simulated wide-band EIS curves from three-dimensional (3D) finite element modeling (FEM). Then, equivalent volume fraction applicable to the growing cell was extracted from the ECM for the geometry-dependent sensing characteristics of the EIS sensing unit. Subsequently, experimental verification of the developed ECM was performed by measuring the wide-band EIS signals of an individual cell. Afterwards, a lumped parameter model (LPM), incorporating the ECM of the microfluidic device and a simplified model of the peripheral circuit in the EIS measurement system, was established for the entire EIS measurement system. Finally, long-term EIS measurements were analyzed to examine the relationship between wide-band EIS signals and the geometric parameters of a growing cell.

## 2. Materials and Methods

### 2.1. Design Concept of EIS Sensing Unit

**Figure 1:**
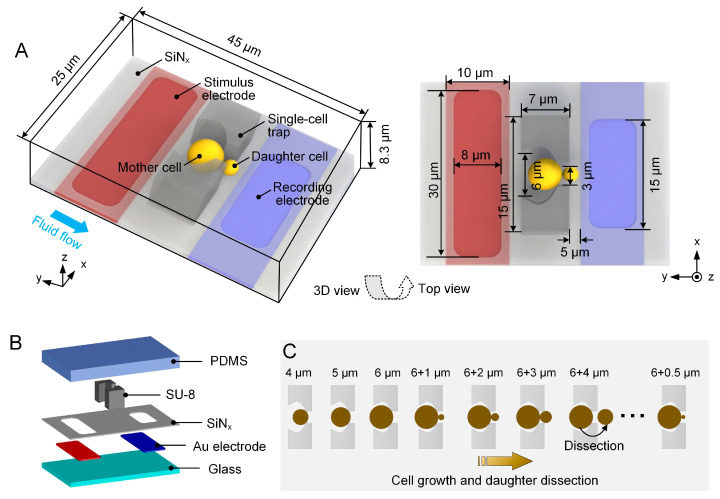
Schematics of in situ EIS monitoring of individual budding yeast cells in a sensing unit. (**A**) Three-dimensional view and top view of the EIS sensing unit. The single yeast cell (yellow) is trapped, cultured, and measured in the single-cell trap made of SU-8 photoresist. The immobilized mother cell grows and produces daughter cells in the downstream orifice. For the EIS measurement, an AC signal is applied to the stimulus electrode (red) and the induced current is measured through the recording electrode (blue). (**B**) Exploded view of the EIS sensing unit. It consists of a glass substrate, a pair of gold (Au) coplanar microelectrodes, a SiNx insulation layer, and a capture-culturing dissection structure made of SU-8 photoresist. (**C**) Schematic illustration of the budding process of an individual yeast cell within the trap. The mother cell first grows into a critical size from 4.0 μm to 6.0 μm before giving birth to its first bud. Then, the bud continuously grows in the downstream opening, with a diameter increasing from 1.0 μm to 4.0 μm until dissection.

The design concept of the EIS sensing unit is based on our previous work, which facilitates the immobilization, long-term culturing, and EIS measurement of a single budding yeast cell [22,23]. Figure 1A shows the schematics of the unit, comprising a glass substrate, a pair of coplanar microelectrodes, a silicon nitride (SiN_x_) passivation layer, and an 8 µm high SU-8 cell-trapping microstructure. The SiN_x_ layer is patterned onto the electrodes and then is etched to open rectangular windows so that the sensing region of electrodes can be exposed to the cell-culturing medium. The microstructure features two pillars facing each other, together forming a bowl-shaped wide opening upstream and a narrow orifice downstream, thus enabling single-cell immobilization (Figure 1B).

During long-term cell culturing, the medium continuously flows through the opening, ensuring reliable maintenance of immobilized yeast mother cells. The mother cell first grows to a critical size before generating its first bud. Subsequently, the daughter cell continues to proliferate within the downstream opening and can be effectively dissected after the completion of each cell cycle, followed by the emergence of a new bud (Figure 1C). Meanwhile, EIS measurements are undertaken by applying an alternating current (AC) voltage with a swept frequency from 1 kHz to 1 MHz to the 30 μm wide stimulus electrode upstream and collecting the response current through the 15 μm wide recording electrode downstream. The response current, which is subsequently converted to the form of amplitude and phase, can elucidate various cellular activities such as cell growth, bud emergence, and daughter-cell dissection.

### 2.2. FEM of the EIS Sensing Unit

To determine the initial parameters of the ECM, 3D FEM of the EIS sensing unit was firstly established in COMSOL Multiphysics (COMSOL Inc., Stockholm, Sweden). As illustrated in Figure 1A, the sensing unit is placed in a cuboid domain with a 45 μm × 25 μm × 8.3 μm outer profile. Residual space within the domain is filled with cell-culturing medium. The cell placed in the sensing unit is set as a sphere with its diameter ranging from 4 μm to 6 μm at an interval of 0.5 μm. When simulating an EIS measurement of polystyrene beads, a 6 μm diameter insulated sphere was employed.

Electric conductivity (*σ*) and relative permittivity (*ε_r_*) of the materials used in simulation, including glass, Au, SiN_x_, SU-8, medium, and polystyrene bead and cell, are listed in Supplementary Table S1. The cell was simplified from a four-shelled sphere into a single-shelled homogeneous spherical model to mimic the complex dielectric properties of an individual budding yeast cell. The relative permittivity (*ε_c_*) and electric conductivity were considered as a function of the cell diameter (*D*) and were calculated over a frequency range of 10 kHz–10 MHz in our previous work [22]. A simplified 0.5 μm thick double-layer capacitance at the electrode–electrolyte interface, which maintains the same specific contact impedance, was modeled using the equation in the previous work [22,24].

To perform EIS simulation, the “Electric Currents” physics from the AC/DC Module in COMSOL was applied to solve the current conservation equation based on Ohm’s law using electric potential as a dependent variable. Parameter and physics settings in simulation are listed in Supplementary Table S2. The electrical potential of the stimulus and recording electrodes were set to 1 V and 0 V, respectively. A total of 100 equidistant frequency points were selected for sampling within the range of 10 kHz to 10 MHz. The model was subjected to a predefined “Physics-controlled mesh” for the process of meshing. Frequency-domain calculations were performed for the defined models, including an empty EIS sensing unit, a unit with a single bead, and a unit with an individual cell. The calculated current density on the surface of the recording electrode at each sampling point was integrated over the surface area to deduce the response current.

For data normalization, the amplitude and phase of the response current in a pure cell-culturing medium were used as reference values (*A_e_*, *θ_e_*). Consequently, the relative amplitude (*A_r_* = *A*/*A_e_*) and relative phase (*θ_r_* = *θ* − *θ_e_*) were employed to elucidate the impedance changes in the sensing unit when occupied by a cell or a bead.

### 2.3. ECM of the EIS Sensing Unit with a Cell

A typical equivalent circuit model (ECM) of a single cell located between paired electrodes is shown in Supplementary Figure S1. The model is characterized by five electrical components: the resistance of the cell-culturing medium (*R_m_*), the capacitance of the cell-culturing medium *C_m_*, the capacitance of the cell membrane (*C_mem_*), the resistance of the cytoplasm (*R_c_*), and the capacitance of the electric double layer (*C_dl_*). The initial values assigned to each electrical component in the ECM are determined by the size and dielectric properties of the medium and the cell, as well as the geometrical parameters of the microfluidic device. They can be defined by the following equations [16]:

(1) $$R_{m}=\frac{1}{\sigma_{m}\left(1-3\varphi /2\right)G_{f}}$$

(2) $$C_{m}=\varepsilon_{m}\frac{2\varepsilon_{m}+\varepsilon_{c}-2\varphi \left(\varepsilon_{m}-\varepsilon_{c}\right)}{2\varepsilon_{m}+\varepsilon_{c}+\varphi \left(\varepsilon_{m}-\varepsilon_{c}\right)}G_{f}$$

(3) $$R_{c}=\frac{4\left(\frac{1}{2\sigma_{m}}+\frac{1}{\sigma_{c}}\right)}{9\varphi G_{f}}$$

(4) $$C_{mem}=\frac{9\varphi \left(\frac{D}{2}\right)C_{mem,0}}{2}G_{f}$$

where *σ_c_*, *σ_m_*, and *σ_mem_* are the conductivities of the cell cytoplasm, culture medium, and cell membrane, respectively. *ε_c_*, *ε_m_*, and *ε_mem_* are the relative permittivities of the cell cytoplasm, culture medium, and cell membrane, respectively. *D* is the cell diameter. *C_mem_*_,0_ is the capacitance per unit area of the cell membrane, with a reference value of 1.4 × 10^−3^ F/m^2^ [16]. *G_f_* is the geometric constant related to the area of the electrode to the channel length [16]. *φ* is the equivalent volume fraction, which is defined as the volumetric occupation of the cell/bead in the effective sensing region due to the complex distribution of the electric field in the EIS sensing unit. And the effective sensing region is defined by the space where the electric field gradient significantly impacts measurements. The relationship between *φ* and different geometry dimensions of the cell (i.e., scaling exponent of the cell diameter) can be examined by linear regression.

Maxwell’s mixture theory was used to calculate the impedance of the circuit components. The equivalent complex impedance of the sensing unit in Supplementary Figure S1, $\tilde{Z_{mix}}\left(\omega \right)$, is given by the following:

(5) $$\tilde{Z_{mix}}\left(\omega \right)=\frac{\left(R_{m}+\frac{1}{j\omega C_{mem}}\right)\cdot \frac{R_{m}}{1+j\omega R_{m}C_{m}}}{R_{m}+\frac{1}{j\omega C_{mem}}+\frac{R_{m}}{1+j\omega R_{m}C_{m}}}+\frac{2}{j\omega C_{dl}}$$

The simplified form is given by the following:

(6) $$\tilde{Z_{mix}}\left(\omega \right)=\frac{R_{m}(1+j\omega R_{c}C_{mem})}{j\omega R_{c}C_{mem}+(1+j\omega R_{m}C_{m})(1+j\omega R_{c}C_{mem})}+\frac{2}{j\omega C_{dl}}$$

According to the objective function between the response current and impedance $\left|\tilde{I}(f)\right|=\left|1/\tilde{Z}(f)\right|$, lsqcurvefit solver in MATLAB 2023a (MathWorks, Natick, MA, USA) was used in the least-squares fitting by substituting equations from (1) to (4). Evaluation of the fitting was conducted using the goodness-of-fit test and the coefficient of determination *R*^2^.

### 2.4. Experimental Setup and Protocol for EIS Measurement

To perform the long-term culture and EIS measurement of single-immobilized budding yeast cells, a microfluidic device was designed (Supplementary Figure S2A). This device comprises a glass substrate, array-formatted electrode pairs, a SiN_x_ insulation layer, an SU-8-based microchannel, and a polydimethylsiloxane (PDMS) ceiling (Supplementary Figure S2B), and it features a straight microchannel with arrayed bowl-shaped single-cell traps. Pairs of coplanar microelectrodes are patterned into the cell-trap array and are routed to the pads on device edges for electrical connection.

During the long-term cell culturing, EIS measurements were performed by applying frequency-sweeping AC voltage with a fixed amplitude (10 kHz–10 MHz, Vp: 1 V) to the stimulus electrode and acquiring the response current from the recording electrode. To sequentially address and select different electrode pairs, a printed circuit board (PCB) with a multiplexed switching control circuit was designed and connected to the device. Detailed experimental setup is illustrated and described in Supplementary Figure S2C.

A diploid strain of the budding yeast *Saccharomyces cerevisiae*, derived from BY4743, was used in the long-term EIS monitoring of cell growth. Cells were cultured, activated, and inoculated in dextrose complete (SD) medium following standard biological procedures. Experimental protocol, including device sterilization, cell loading, trapping, and long-term culturing, can be referred to in the procedure outlined in our previous work [23]. EIS measurements were initiated once the fluid and EIS sensing system reached stability. Time-lapse EIS measurements were performed, while bright-field images were taken at a 2 min interval for over 45 min simultaneously.

### 2.5. LPM of the Entire EIS Measurement System

To perform equivalent circuit modeling of the EIS measurement of a single cell, a lumped parameter model (LPM) for the entire EIS measurement system was established. This LPM includes the ECM of the microfluidic device and a simplified model for the peripheral circuit. The peripheral circuit, designed for impedance detection of the arrayed EIS sensing unit, consists of conductors, gating circuits, and transimpedance amplifiers. Supplementary Figure S3 illustrates a complete LPM for the EIS sensing unit with a single mother cell (denoted as Device Under Test, DUT) and the entire EIS sensing system. The microfluidic device for EIS sensing is encapsulated and communicates with the instrument via PCB boards and coaxial cables. Parasitic capacitance arises from conductive pathways on the PCB traces, IC chips, pads, vias, and coaxial cables.

Parameters in the LPM (including *V_in_*, *V_out_*, *C_p_*, *L_w_*, *R_w_*, *C_ac_*, and *R_ac_*) along with their respective values are detailed in Supplementary Table S3. Notably, the wire inductance (*L_w_*) is typically in a microhenry (μH) range at frequencies varying from 10 kHz to 10 MHz. The wire resistance (*R_w_*) is typically less than 100 Ω. As a result, the seriesly connected *L_w_* and *R_w_* could be considered negligible when the impedance is significantly lower than the expected impedance of the DUT. Furthermore, it is assumed that the parasitic capacitance *C_p_* in both of the stimulus loop (from *V_in_* to the DUT) and the recording loop (from the DUT to *V_out_*) are identical.

To establish an LPM, the response current of the EIS sensing system was measured with fixed resistors. Additionally, the microfluidic device, when loaded with a pure cell-culturing medium and cells, was incorporated into the circuit as DUTs. The genetic algorithm (GA) in MATLAB was utilized to fine-tune the parameters in the LPM so that they aligned with the experimentally obtained data. The specific settings for the GA algorithm are detailed in Supplementary Table S4.

## 3. Results

### 3.1. FEM Simulation of EIS Measurement of Cell Growth

**Figure 2:**
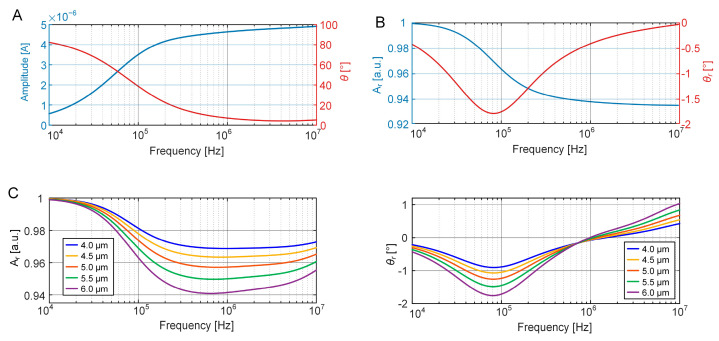
FEM simulation of EIS measurement of the sensing unit. (**A**) Raw amplitude (*I_e_*) and phase (*θ_e_*) of the response current of an empty EIS sensing unit over a frequency range from 10 kHz to 10 MHz. (**B**) Relative amplitude (*A_r_* = *I*/*I_e_*) and phase (*θ_r_* = *θ* − *θ_e_*) induced by the presence of a bead in the EIS sensing unit. (**C**) Relative amplitudes and phases of a growing mother cell modeled by increasing its diameter from 4.0 μm to 6.0 μm at an interval of 0.5 μm.

To determine the initial parameters and accurately fit the circuit components in the equivalent circuit model, FEM simulations for the EIS characterizations of an empty EIS sensing unit, and the unit occupied with a single bead and cell were performed. Numerical calculations were performed to determine the raw amplitudes and phases of response currents over frequencies ranging from 10 kHz to 10 MHz. As illustrated in Figure 2A, the presence of a double-layer capacitor at the electrode–electrolyte interface results in an increase in the response current amplitude with rising frequencies, while the phase decreases concomitantly. Then, the EIS characterization of a bead in the sensing unit was performed through the relative amplitude (*A_r_*) and relative phase (*θ_r_*), using the raw data from the empty unit as a reference.

As shown in Figure 2B, the relative amplitudes maintain a value of approximately 1 when the frequency is below 10 kHz. This is because, at low frequencies, impedance primarily depends on the double-layer capacitance. On the contrary, in frequencies exceeding 10 kHz, the presence of a bead results in an increase in the impedance compared to an empty unit. Consequently, there is a decrease in both the relative amplitudes and phases of the response current. Moreover, with increasing frequency, the influence of the capacitive components on the impedance diminishes. The insulating nature of the bead becomes more pronounced, resulting in reduced phase alternations. Hence, the relative phase reaches its minimum value at approximately 80 kHz.

EIS characterization of cell growth in the sensing unit was performed by increasing the cell diameter from 4 μm to 6.0 μm at an interval of 0.5 μm. Figure 2C shows that the relative amplitudes have minor difference at frequencies below 100 kHz. When the frequency increases, the differences in *A_r_* are significant between 500 kHz and 1 MHz. In addition, cell growth leads to a corresponding rise in impedance, thereby reducing the amplitude. The relative phase also decreases with increasing cell size, and the difference is more significant near 80 kHz. On the other hand, at a high frequency (>1 MHz), this decreasing trend is reversed, and the increase in cell size leads to a rise in the relative phase. This is due to the gradual dominance of the cytoplasmic resistance at a high frequency.

### 3.2. Establishment of the ECM of the EIS Sensing Unit

The ECM of the EIS sensing unit includes the unsolved components of medium resistance (*R_m_*), medium capacitance (*C_m_*), cell membrane capacitance (*C_mem_*), cytoplasmic resistance (*R_c_*), double-layer capacitance (*C_dl_*), and the geometric constant (*G_f_*).

The initial step involved fitting the equivalent impedance to the simulated EIS data of the empty sensing unit. The parameters, *C_mem_*, *R_c_*, and the volume fraction, *φ*, were all set to 0. Consequently, Equations (1) and (2) are simplified to the following:

(7) $$R_{m}=\frac{1}{\sigma_{m}G_{f}}$$

(8) $$C_{m}=\varepsilon_{m}G_{f}$$

After being substituted into Equation (6), *G_f_* and *C_dl_* were calculated using the modified equation of equivalent impedance:

(9) $$\tilde{Z}(\omega )=\frac{1}{\left(\sigma_{m}+j\omega \varepsilon_{m}\right)G_{f}}+\frac{2}{j\omega C_{dl}}$$

**Figure 3:**
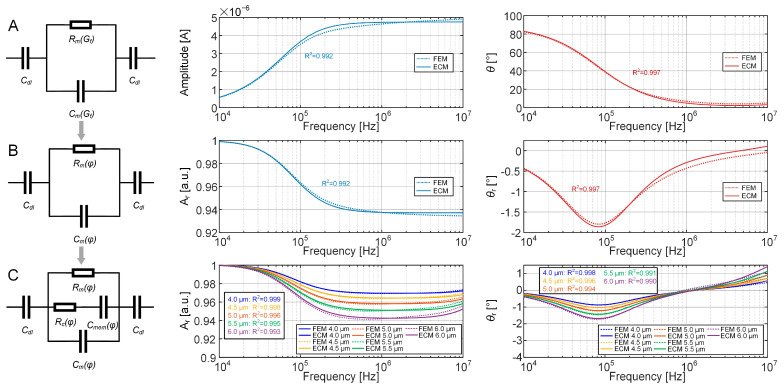
ECM of the EIS sensing unit. (**A**) ECM and its fitting curves with EIS data derived from FEM simulation of the pure culture medium over a frequency range from 10 kHz to 10 MHz. The equivalent circuit model, along with the circuit elements to be solved (*C_dl_* and *G_f_* in *C_m_* and *R_m_*), is depicted on the left side of the fitted curve. In the fitted amplitude and phase curves, the solid lines represent EIS data from the FEM simulation, while the dashed lines illustrate the curves obtained from calculated equivalent circuit elements. (**B**) ECM and its fitting curves with relative amplitude and phase derived from FEM simulation of the polystyrene bead. The ECM with the circuit elements to be determined (*φ* in *C_m_* and *R_m_*) is depicted on the left. (**C**) ECM and its fitting curves with relative amplitude and phase derived from FEM simulation of the growing mother cell with increasing diameter from 4.0 μm to 6.0 μm at an interval of 0.5 μm. The ECM with the circuit elements to be determined (*φ* in *C_m_*, *R_m_*, *C_mem_*, and *R_c_*) is depicted on the left.

Curves of the amplitude and phase of the empty sensing unit are shown in Figure 3A, along with the derived values for *G_f_* and *C_dl_* (*G_f_* = 5.280 × 10^−6^ m, *C_dl_* = 1.844 × 10^−11^ F). The coefficients of determination (*R*^2^) for both the amplitude and phase confirm fine goodness of fitting.

Considered as an insulator, the polystyrene bead introduces an equivalent volume fraction (*φ*) into the sensing unit. With the determined *G_f_* and *C_dl_*, *C_m_* and *R_m_* in Equations (1) and (2) reduce to the following:

(10) $$R_{m}=\frac{1}{4.752\times 10^{-6}\times (1-1.5\varphi )}$$

(11) $$C_{m}=\frac{6.93-6.67\varphi }{165+78\varphi }\times 10^{-2}$$

Substituting the determined *G_f_* and *C_dl_* and Equations (10) and (11), the impedance of the sensing unit ($\tilde{Z}(\omega )$) reduces to the following:

(12) $$\tilde{Z}(\omega )=\frac{165+78\varphi }{\left(1-1.5\varphi \right)\left[\left(165+78\varphi \right)+j\omega \times 2100.7\times \left(6.93-6.67\varphi \right)\right]}+\frac{1}{j\omega \times 9.23\times 10^{12}}$$

The optimal solution for the equivalent volume fraction (*φ*) was calculated to be 0.042% when Equation (10) was solved using the EIS simulation of the sensing unit occupied with a bead, resulting in a high coefficient of determination, as shown in Figure 3B.

The equations for the EIS sensing unit occupied with a single cell introduces *C_mem_* and *R_c_* into the ECM using the following equations:

(13) $$R_{c}=\frac{1.2224+\frac{1}{\tilde{\sigma }(D)}}{4.752\times 10^{-5}\times \varphi }$$

(14) $$C_{mem}=119.5\times \varphi \times D$$

With the substitutions of *G_f_* and *C_dl_* and Equations (10), (11), (13) and (14) into Equation (6), the optimal solutions for the equivalent volume fraction (*φ*) were determined for each cell diameter (*D* = 4, 4.5, 5, 5.5, and 6 μm) in Table 1. The fitted curves of *A_r_* and *θ_r_* corresponding to the cell with different diameters are plotted in Figure 3C, exhibiting high values of *R*^2^.

The amplitude and phase curves derived from the ECM fittings of the empty sensing unit, as well as the unit occupied with a single bead and a growing cell, exhibit considerable alignment with FEM data in the mid-to-low-frequency range. However, the precision of these fittings tends to decrease in higher frequencies.

### 3.3. Equivalent Volume Fraction of the Growing Cell

**Figure 4:**
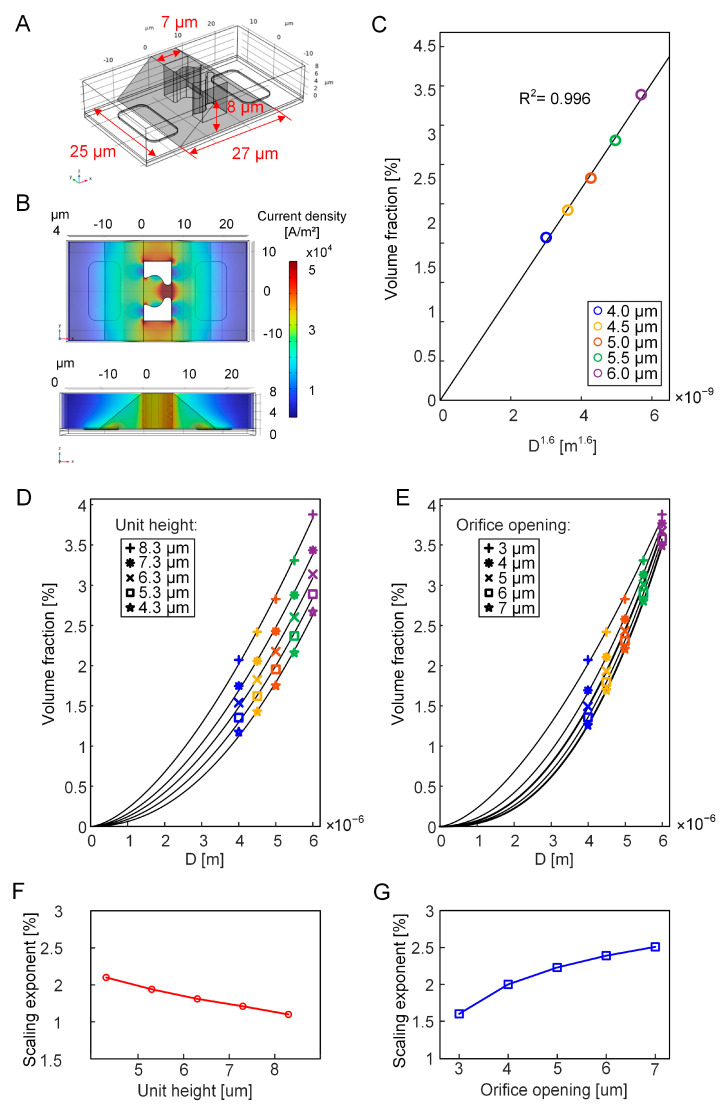
Equivalent volume fraction of the bead and the growing cell. (**A**) Schematics of the sensing region (the prism shaded in gray) for the EIS sensing unit. (**B**) Current density distribution in the FEM for the sensing unit. (**C**) Linear relationship between the equivalent volume fraction and the cell diameter raised to 1.6th power for varying cell sizes. The cell diameter ranges from 4.0 μm to 6.0 μm at a 1 μm interval. (**D**) Equivalent volume fraction against cell diameter for varying height of the single-cell trap, ranging from 4.3 μm to 8.3 μm at a 1 μm interval. (**E**) Equivalent volume fraction against cell diameter for different orifice openings, from 3 μm to 7 μm at a 1 μm interval. (**F**,**G**) Scaling exponents for the linear relationships between the equivalent volume fractions and cell sizes across different heights of the unit and various opening widths of the orifice, respectively.

The equivalent volume fraction (*φ*) of the bead is determined by the ratio of the bead volume to the effective sensing region, as derived from Equation (10). The volume of the bead can be directly calculated by the geometric sphere equation V = 4πR^3^/3. The effective sensing region, defined by the space where the electric field gradient significantly impacts measurements, was estimated based on the distribution of current density through finite element modeling. As shown in Figure 4A,B, a prism with a volume of 3400 μm^3^ was selected, where the current density exceeds 2.5 × 10^4^ A/m^2^. After subtracting the region of the SU-8-trapping structure from the selected prism, the equivalent volume fraction of the bead is calculated to be 0.039%. The result that refers to the geometric modeling agrees well with the analytical value, which is 0.042%. When transitioning from an insulated bead to a growing cell, the equivalent volume fraction *φ* that corresponds to different cell diameters in Table 1 is reasonably ranged between 0 and 0.042%.

Ideally, in the IFC sensing system, cells are fully exposed to the electric field, resulting in a robust correlation between impedance data and cell volume. Moreover, our previous study has demonstrated that there is a significant linear relationship between impedance and the height (1D) or effective area (2D) of a cell immobilized at a small orifice in an EIS sensing microfluidic device. However, when the orifice opening exceeds the cell diameter, the impedance demonstrates a linear fit with the volume (3D) [21].

Given the direct relationship between the impedance of the sensing unit and the equivalent volume fraction, it is logical to deduce that changes in the equivalent volume fraction should correspond proportionally to variations in cell diameter and the effective area of volume. However, Supplementary Figure S4 demonstrates that the equivalent volume fraction does not have a linear relationship with the cell diameter or volume. Instead, it follows a power-law function, based on the single-cell diameter:

(15) $$\varphi =k{\cdot D}^{a}$$

where *k* is the fitting parameter, *D* is the cell diameter, and *a* is the scaling exponent to keep a linear relationship between the equivalent volume fraction and the cell diameter. As shown in Figure 4C, the optimal geometric dimension exhibiting a linear correlation with the equivalent volume fraction corresponds to the 1.6th power of the cell diameter.

The exponent linearly associated with the equivalent volume fraction ranges between one and three. Such a relationship can be attributed to the configuration of the bowl-shaped single-cell trap and its associated microelectrodes. The electric current is directed to flow through both the wide opening and the narrow orifice of the trap. Consequently, the effective sensing region between the electrodes becomes highly sensitive to the cross-sectional area of the trap.

To further explore the geometry-dependent sensing characteristics, the height of the single-cell trap was adjusted from 8.3 μm to 4.3 μm at an interval of 1 μm. The geometric dimensions that linearly correlated to the equivalent volume fraction were obtained by fitting the ECM to FEM across different trap heights (Supplementary Figure S5). As a result, the scaling exponent rises from 1.6 to 2.1, consistently yielding a high coefficient of determination above 0.996 (Figure 4D,F). Then, the width of the orifice opening was expanded from 3 μm to 7 μm at an interval of 1 μm (Supplementary Figure S6). Even when the orifice width exceeded the cell diameter, the cell remained fixed at the orifice in the finite element modeling. This change leads to a rise in the scaling exponent to 2.51, accompanied by high values of R^2^ (Figure 4E,G). Both reducing the height and expanding the orifice results in an increase in the scaling exponent. This suggests that when a cell is captured at a lower trap with a wider orifice, more electric current would flow through the surrounding space rather than the cell. As demonstrated in our previous work [21], the IFC system exhibits high sensitivity to sample volume, and the impedance signals are linearly correlated to sample height (1D) and effective area (2D). Therefore, decreasing height and increasing orifice width would make the EIS sensing unit behave more like an IFC sensing system. Moreover, compared to the single-cell trap design in our previous work [21], the trap design presented in this work constrains current not only at the orifice but also within the bowl-shaped space. This sophisticated design results in an equivalent volume fraction that does not correspond directly to either cell diameter (1D) or effective area (2D).

### 3.4. LPM Fitting of the Entire EIS Measurement System

**Figure 5:**
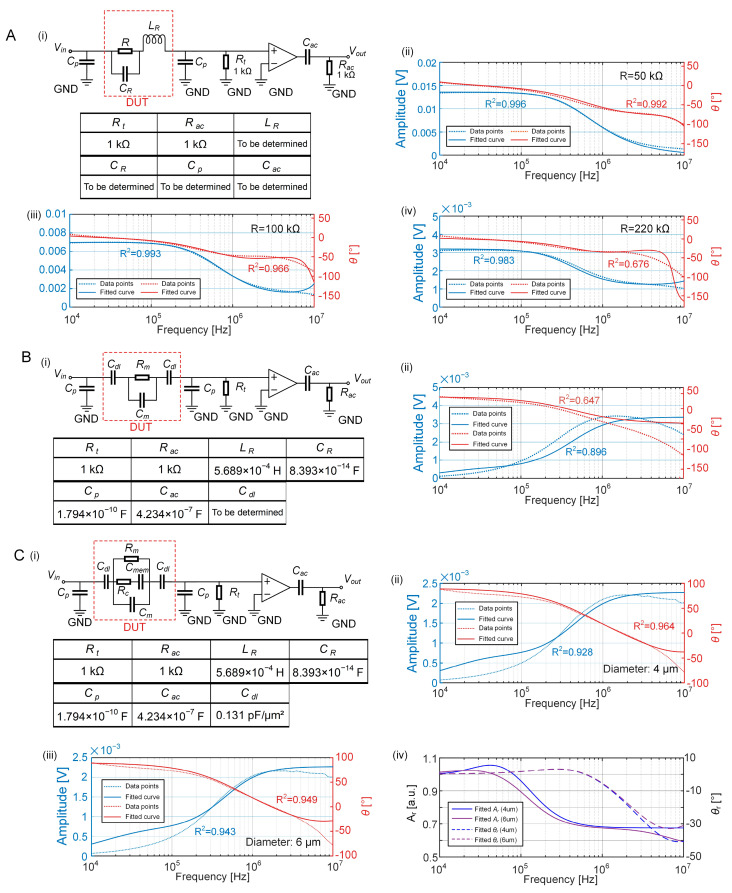
LPM of the entire EIS measurement system. (**A**) LPM for the peripheral circuit using fixed resistors (i) and its fitting curves using the optimized solutions for parameters of *L_R_*, *C_R_*, *C_p_*, and *C_ac_* (ii: R = 50 kΩ, iii: R = 100 kΩ, iv: R = 220 kΩ). In the fitted amplitude and phase curves, the solid lines represent impedance data measured through the EIS sensing system, while the dashed lines illustrate the curves derived from calculated equivalent circuit elements. (**B**) LPM for the microfluidic device filled with a pure medium (i) and the fitting curves using the optimized solutions for parameters of *C_dl_*, *C_m_*, and *R_m_* (ii). (**C**) LPM for the microfluidic device capturing a growing single cell (i) and its associated fitting curves compared to measured results (ii: diameter = 4 μm, iii: diameter = 6 μm). (iv) Relative amplitude (solid lines) and phase (dashed lines) of LPM for the mother cell with diameters of 4 μm (blue) and 6 μm (purple).

To obtain the electrical components in the lumped parameter model for the peripheral circuit, fixed resistors with the values of 50 kΩ, 100 kΩ, and 220 kΩ were initially incorporated into the circuit as the DUT. The response currents of the EIS sensing system were measured. As shown in Figure 5A(i), an analytical function for *V_out_* was formulated in MATLAB using parameters *L_R_*, *C_R_*, *C_p_*, and *C_ac_* for determination. The fitted curves of amplitudes and phases of the response currents were calculated, as shown in Figure 5A(ii–iv). Notably, the LPM fitting curves for the 50 kΩ and 100 kΩ resistors yield high *R*^2^ values, indicative of a good fit. Conversely, the LPM fitting for the 220 kΩ resistor shows poor alignment in the high-frequency range of the phase curve. This discrepancy may potentially be attributed to the increased tolerance typically associated with larger resistance values, which may have introduced errors in the fitting process.

The microfluidic device perfused with a pure medium was then evaluated as the DUT in the EIS measurement system. Concurrently, the amplitude and phase curves were recorded (Figure 5B). The DUT was conceptualized as an electrical circuit consisting of a double-layer capacitance (*C_dl_*), a medium capacitance (*C_m_*), and a medium resistance (*R_m_*), based on the ECM of an empty EIS sensing unit. Using the deduced solutions of *L_R_*, *C_R_*, *C_p_*, and *C_ac_*, the double-layer capacitor *C_dl_* was determined to be 0.131 pF/μm^2^ through the optimized solution fitting to *V_out_*. However, the amplitude and phase of the fitting curves for this LPM model do not match well in the mid-to-high-frequency range. This discrepancy may be attributed to the heightened significance of parasitic inductance, parasitic capacitance, and other factors in the peripheral circuit at high frequencies, which were simplified in the design of the model.

Ultimately, the microfluidic device with single-cell-trapping was used as the DUT in the EIS measurement system. With the established ECM of a single cell immobilized in the EIS sensing unit, parameters such as the medium capacitance (*C_m_*), medium resistance (*R_m_*), cytoplasm resistance (*R_c_*), and cell membrane capacitance (*C_mem_*) were obtained by fitting them to the tested data points of cells with the diameters of 4 μm and 6 μm. Optimized solutions for *C_m_*, *R_m_*, *R_c_*, and *C_mem_* are listed in Table 2. A significant difference is observed in the relative amplitude of cells with 4 μm and 6 μm diameters at 1 MHz (Figure 5C). Due to the increased size, the amplitude of the cell with a 6 μm diameter was found to be smaller than that of the cell with a 4 μm diameter.

**Figure 6:**
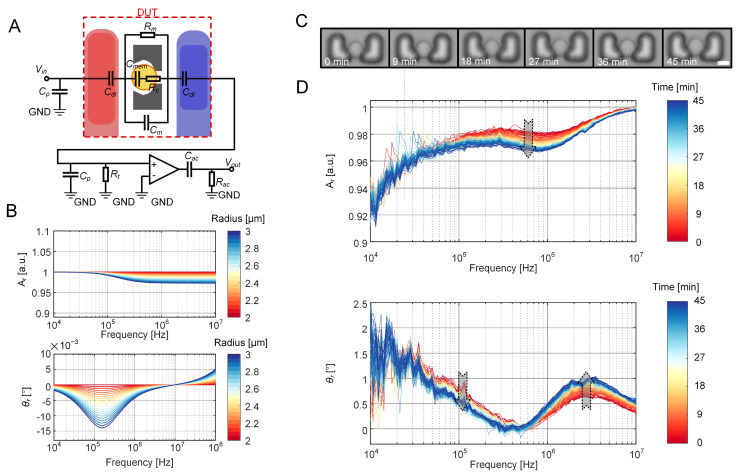
EIS measurements of single yeast growth. (**A**) LPM of the entire EIS measurement system when an individual cell occupies the EIS sensing unit. (**B**) Relative amplitudes and phases of a growing cell, with diameters ranging from 4 μm to 6 μm (increment = 0.05 μm), derived using the LPM of the EIS measurement system. Each curve represents the relative amplitude and phase corresponding to each cell size. (**C**) Time-lapse bright-field images showing the growing process of a single yeast cell in the sensing unit within 45 min. Scale bar: 2 μm. (**D**) Time-lapse relative amplitudes and phases of a growing mother cell over a 45 min EIS measurement period. Each curve corresponds to the relative amplitude and phase of the cell impedance measured at different time points.

The conclusion was drawn that the equivalent volume fraction of a cell is proportional to the 1.6th power of the cell diameter. This relationship was used to interpolate and determine the cytoplasm resistance (*R_c_*) and cell membrane capacitance (*C_mem_*) of cells with diameters ranging from 4 μm to 6 μm in increments of 0.05 μm. Upon deriving all parameters in the LPM of the entire EIS measurement system, the relative amplitude and phase were calculated to simulate the electrical response of cell growth (Figure 6A). Figure 6B shows that as the diameter expands, the cytoplasm resistance of the cell decreases while the cell membrane capacitance increases. The decrease in relative amplitudes can be attributed to the increase in cell size, which progressively occupies a larger volume of the culture medium. Given that the cell conductivity is lower than that of the culture medium, the response current inevitably decreases. Moreover, an increase in cell membrane capacitance leads to a continuous decrease in phase.

The variations in complex impedance induced by the growth of the mother cell were experimentally validated. A virgin budding yeast cell was identified through confocal microscopy imaging, and its growth process was recorded. Concurrently, time-lapse EIS measurements were conducted for approximately 45 min. As shown in Figure 6C, the most significant difference can be observed in the relative amplitude reduction in the mid-frequency (500 kHz to 2 MHz). Additionally, the relative phase shows a decrease near 100 kHz and a notable inverse trend in the higher frequency range (1 MHz to 10 MHz). An increase in cell size correspondingly augments cytoplasmic resistance and cell membrane capacitance, leading to an amplitude rise and a phase decrease within the mid-frequency range. Moreover, as frequency increases, the dominance of the cell membrane capacitor gradually diminishes, while the influence of the cytoplasm and vacuoles intensifies, resulting in an overall enhancement in cell conductivity

Long-term variations in relative amplitudes and phases exhibit a similar trend with the calculated results of the LPM in the low- and mid-frequency range. However, the relative phases derived from the LPM show an inverse increase in the higher frequency (>10 MHz) compared to the curves obtained from EIS measurements. This discrepancy in high-frequency performance could be attributed to an oversimplified equivalent circuit that does not adequately account for high-frequency components.

## 4. Discussion

This study presents the establishment of equivalent circuit models to characterize single-cell growth using electrical impedance spectroscopy (EIS). Initially, 3D FEM was employed to simulate the in-situ impedance measurement of the EIS sensing unit, both in its empty state and when it contained either a single bead or a single budding yeast cell. Numerical calculations of current responses over a frequency range from 10 kHz to 10 MHz were conducted for various cell growth stages. Next, an equivalent circuit model for the immobilized cell was developed by fitting electrical responses with the results from FEM, allowing for the determination of typical electrical components in the ECM. The equivalent volume fraction applicable to the growing cell was extracted from the ECM for the geometry-dependent sensing characteristics of the EIS sensing unit, as well as its different geometrical configurations. Subsequently, a microfluidic device integrated with a microfabricated electrode array and a custom-designed peripheral circuit was used to extract electrical parameters. These parameters were then fitted to establish a lumped parameter model for the entire EIS measurement system. Finally, time-lapse EIS measurements of both empty and cell-occupied sensing units were conducted, confirming the consistency of the proposed equivalent circuit model with impedance changes induced by cell growth.

This study presents the inaugural modeling and analysis of dynamically changing cells in microfluidic EIS characterization along with the entire peripheral circuits using an equivalent circuit. The proposed ECM and LPM effectively replicate the electrical characteristics of the impedance measurement system across a broad frequency range, thereby ensuring accurate prediction of impedance alterations during cellular growth. Moreover, the introduction of a dimensionally reduced equivalent volume fraction was successfully utilized to characterize cellular activities, such as yeast cell growth. The established correlation between the equivalent volume fraction and cell size facilitates the interpolation prediction of the continuous change in cell impedance induced by cell growth. The results are in good agreement with the experimental results in terms of replication and phase curve trends. The proposed geometry-dependent sensing characteristics are intrinsically linked to the physical relationship between the cell and the effective sensing region of the unit, rather than strict analytical derivation. Additional validation of the linear relationship between the suggested scaling law and the equivalent volume fraction could be performed through experimental measurements using microfluidic chips that comprise EIS sensing units with various opening sizes and channel heights.

It is important to note that experimental results may not align perfectly with the fitted ECM and LPM. Specifically, in the high-frequency range, phase variations observed in both electrical impedance simulations and experimental measurements show an inverse trend, where the phase increases as cell volume expands. In contrast, this phenomenon is observed in higher frequencies in the LPM. Potential causes of these discrepancies include the following: (i) Over-simplification of the ECM model. The equivalent circuit model of the cell is based on certain simplifying assumptions, whereas the actual electrical properties of the cell are considerably more complex. For instance, factors such as resistance, capacitance, and membrane conductance can vary depending on environmental conditions. (ii) Ignorance of high-frequency effects in the peripheral circuit. In developing the LPM circuit, several detailed high-frequency effects of the peripheral circuit were not considered, including the non-linear effect of circuit components, high-frequency parasitic capacitance, and issues related to electromagnetic interference and electromagnetic compatibility. In addition, to improve the accuracy of the model in a high-frequency range, it is necessary to enhance the quality of impedance data obtained from EIS measurements, thereby providing more accurate guidance for LPM fitting. The necessary improvements include the following: (i) Improvements in peripheral circuits and measurement setups, including the selection of PCB substrates and optimization of routing. These improvements will contribute to a reduction in signal loss and interference. (ii) Transitioning the connection between the PCB and the microfluidic chip. Fixed connections, such as wire bonding or standardized high-frequency connectors, will ensure the repeatability and stability of signal reading and reduce the random contact impedance brought by the spring-probe connections in the experiments.

Currently, there are various electrical characterization methods at the single-cell level, such as dielectric spectroscopy or impedance tomography. Dielectric spectroscopy, which facilitates swift, high-throughput examinations of the polarization responses of cell populations across various frequencies, exhibits shorts in the comprehensive analysis of various physiological states of the cells in the long term. The advantage of impedance tomography lies in its imaging capability, which allows for the reconstruction of cross-sectional or even three-dimensional information of the target object into images. However, the application has been challenged in resolution and throughput, especially in single-cell resolution. These methods use similar principles to single-cell in situ EIS measurements, but they employ distinct methods of administering electric fields to cells. Therefore, the proposed ECM has the potential to be expanded for use in a variety of methods employing cellular electrical measurements, including dielectric spectroscopy and impedance tomography. For example, the cell was simplified from a four-shelled sphere into a single-shelled homogeneous spherical model in FEM, with calculated complex dielectric properties. The theoretical basis for dielectric spectroscopy can be established by restoring the simplified dielectric parameters of cells to their original form and determining their relationship with electrical impedance spectroscopy. In addition, the single-cell equivalent circuit model can assist the electrical impedance tomography technology to extract the structural characteristics of cells at different frequencies, including the thickness of the cell membrane, morphological changes, and help to provide data analysis for the real-time monitoring of dynamic changes in cells in different biological processes (such as division, proliferation, apoptosis).

In conclusion, this study has demonstrated that the proposed equivalent circuit model is capable of characterizing single-cell growth. With further optimization and development in terms of throughput and long-term data processing, the model and the microfluidic device could potentially offer an automatic and non-optical platform for yeast replicative aging studies and replicative lifespan determination. Additionally, the single-cell in situ EIS measuring platform exhibits extended clinical applications in non-optical monitoring for cell therapy research, diagnostics, drug development, and drug resistance assessment, as well as industrial use in metabolism monitoring for optimization in feeding strategy.

## Figures and Tables

**Table 1:** Optimal solutions for equivalent volume fraction *φ* corresponding to different cell diameters.

*D* [μm]	4	4.5	5	5.5	6
*φ* [%]	0.0207	0.0242	0.0283	0.0331	0.0388

**Table 2:** Solutions for parameters in LPM using cell as DUT.

Diameter [μm]	*C_m_*	*R_m_*	*R_c_*	*C_mem_*
4	997 kΩ	0.98 pF	1.75 MΩ	0.13 pF
6	991 kΩ	0.78 pF	1.09 MΩ	0.20 pF

## Data Availability

Dataset available on request from the authors upon reasonable request.

## Supplementary Materials

The following supporting information can be downloaded at https://www.mdpi.com/article/10.3390/bios15020113/s1, Supplementary Table S1: Electric conductivity and relative permittivity of materials used in FEM simulation. Supplementary Table S2: Parameters and physics settings used in FEM simulation. Supplementary Figure S1: A typical equivalent circuit model (ECM) of a single cell located between a pair of electrodes. Supplementary Figure S2: Detailed experimental setup of the microfluidic device and the connection of instruments. Supplementary Figure S3: A complete lumped parameter model (LPM) for the EIS sensing unit with a single mother cell (denoted as Device Under Test, DUT) and the entire EIS sensing system. Supplementary Table S3: Parameters and their corresponding values used in LPM. Supplementary Table S4: Parameter settings of the Genetic Algorithm. Supplementary Figure S4: Linear fitting of (A) cell diameter and (B) cell volume with the equivalent volume fraction. Supplementary Figure S5: Linear fitting of different geometric dimensions with the equivalent volume fraction across different trap heights (from 4.3 μm to 8.3 μm at a 1-μm interval).
