# Supplementary material for: Equivalent Circuit Modeling and Analysis for Microfluidic Electrical Impedance Monitoring of Single-Cell Growth
Source: Biosensors (Basel). 2025 Feb 14;15(2):113. doi: 10.3390/bios15020113 (PMC11853229; doi:10.3390/bios15020113)
Supplement: Supplementary file 1 [file biosensors-15-00113-s001.zip › biosensors-3443896-supplementary.pdf]

Supplementary Table S1. Electric conductivity and relative permittivity of materials used in FEM simulation

Materials	Electric conductivity σ (S/m)	Relative permittivity ε_r
Glass	1×10^{-14}	4.2
Au	4.56×10^{7}	1
SiN_x	1×10^{-16}	9.7
SU-8	1×10^{-17}	3
Cell culture medium	0.9	81
Polystyrene bead	1×10^{-12}	3
Cell	$\tilde{\sigma}(D)$ [1]	$\tilde{\varepsilon_c}(D)$ [1]
Electrical double layer	$\left(\frac{1}{0.522\times\sqrt{2\pi f}}+0.15\right)\times 5\times10^{-7}$ [1]	$\frac{5\times10^{-7}\times\sqrt{2\pi f}}{0.522}$ [1]

Reference

[1] Geng, Y.; Zhu, Z.; Zhang, Z., et al. Design and 3D modeling investigation of a microfluidic electrode array for electrical impedance measurement of single yeast cells. *Electrophoresis* **2021**, *42*, 1996-2009.

Supplementary Table S2. Parameters and physics settings used in FEM simulation

Simulation parameter	Value
Boundary condition	Electric insulation
Surface	Continuity
Initial values for the electric potential	Default values (0 V)
Potential of the stimulus electrodes	1 V
Potential of the recording electrodes	0 V
Frequency points	100
Sampling range	10 kHz~10 MHz

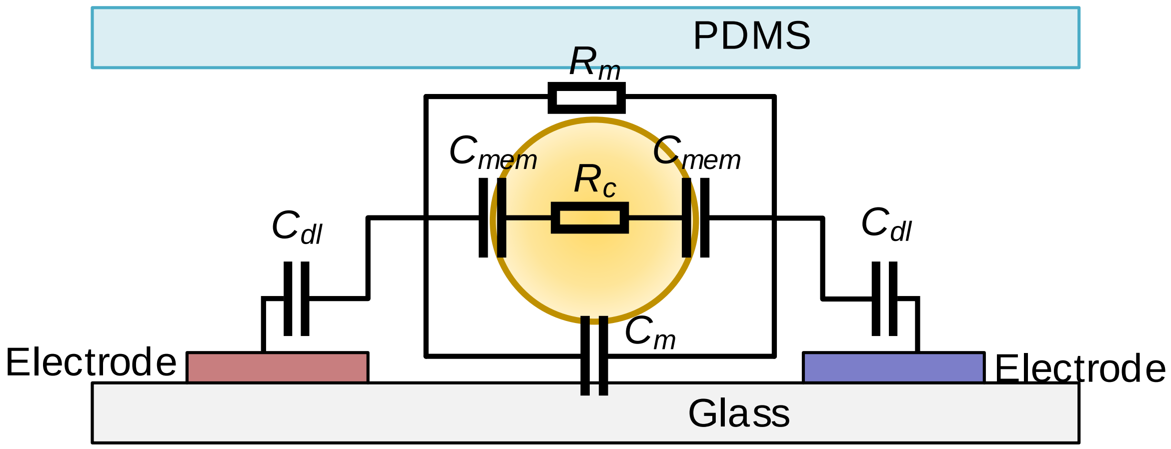

Supplementary Figure S1. A typical equivalent circuit model (ECM) of a single cell located between a pair of electrodes.

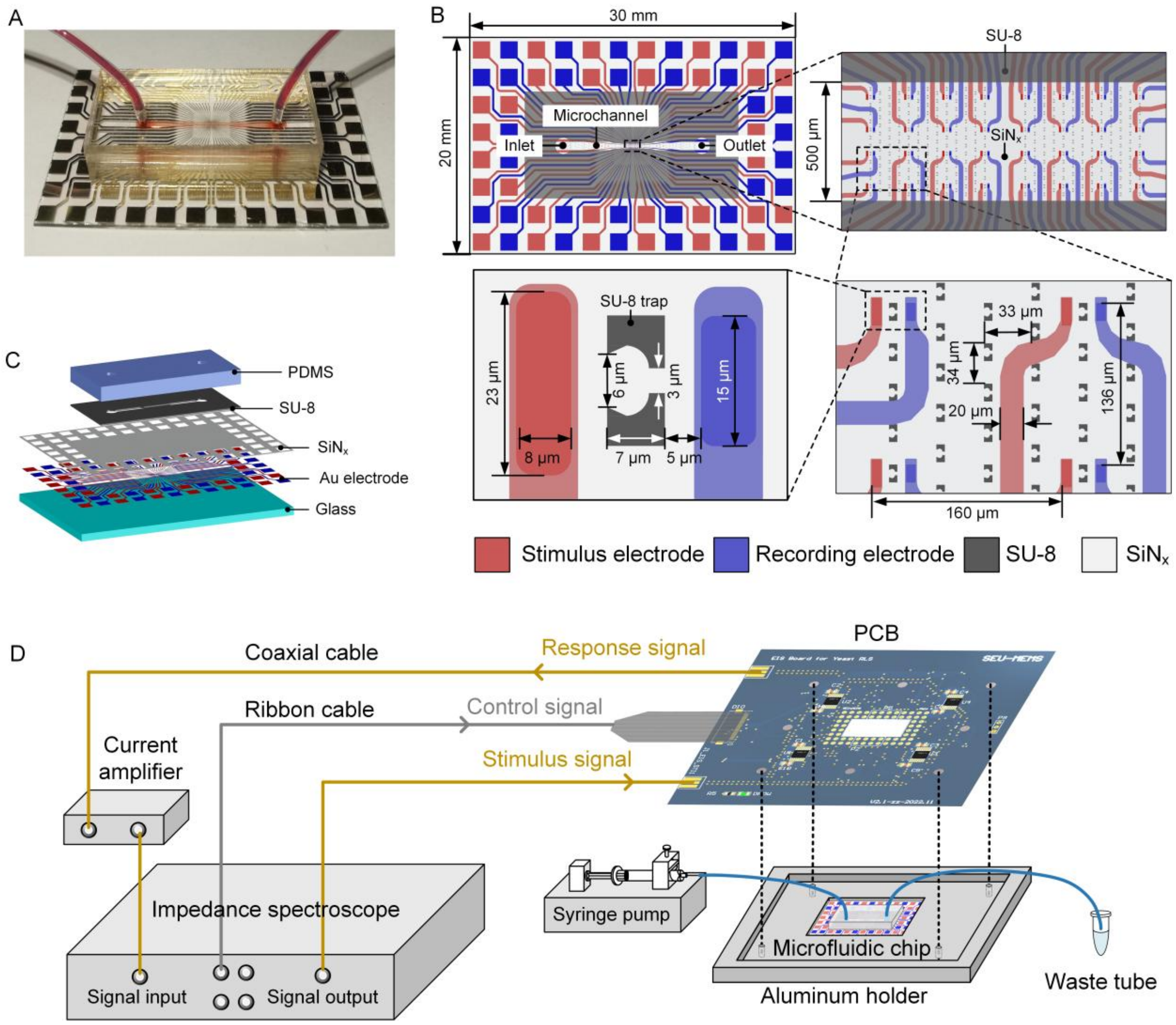

Supplementary Figure S2. Detailed experimental setup of the microfluidic device and the connection of instruments. (A) Photograph of an assembled microfluidic device. (B) Schematics of the microfluidic device with zoom-in view at the microelectrode array and the EIS sensing unit. (C) Exploded view of the microfluidic device. (D) Schematic of experimental setup showing the assembly of device and the connection of instruments. The microfluidic chip was initially clamped between a custom-made aluminum holder and a transparent polymethylmethacrylate (PMMA) cover using plastic screws. Then, the PCB board with spring probes was mounted onto the PMMA cover for electrical connection to the microfluidic chip. Coaxial cables linked the PCB board to the impedance spectroscope (HF2IS, Zurich Instruments, Switzerland; OE2041, Sine Scientific Instrument, China) and the current amplifier (HF2CA, Zurich Instruments, Switzerland; OE4102, Sine Scientific Instrument, China). Polytetrafluoroethylene (PTFE) tubing facilitated the pumping of fluid from a glass syringe attached to a precision syringe pump (neMESYS, CETONI GmbH, Germany) to the chip inlet, and conveyed waste from the outlet to the collection tube. To obtain visual information of cell growth, the aluminum holder was placed on the stage of an inverted confocal microscope (FV3000, Olympus, Japan).

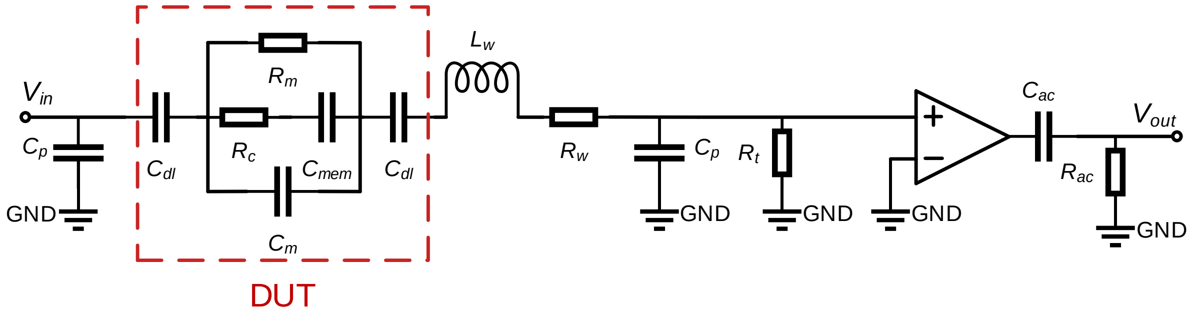

Supplementary Figure S3. A complete lumped parameter model (LPM) for the EIS sensing unit with a single mother cell (denoted as Device Under Test, DUT) and the entire EIS sensing system.

Supplementary Table S3. Parameters and their corresponding values used in LPM

Parameters	Discription	Value
V_{in}	AC stimulus signal	To be determined
V_{out}	Recording voltage	To be determined
C_p	Parasitic capacitance	To be determined
L_w	Wire inductance	< 1 kΩ (negligible)
R_w	Wire resistance	< 100 Ω (negligible)
R_t	Transimpedance	1 kΩ
C_{ac}	AC coupling capacitance	To be determined
R_{ac}	AC coupling resistance	1 kΩ

Supplementary Table S4. Parameter settings of the Genetic Algorithm

**Parameter**	Population size	Constraint tolerance	Function tolerance	Mutation rate	Crossover rate	Maximum generations	Stall generations
**Value**	50	10^{-12}	10^{-12}	0.2	0.5	5000	2000

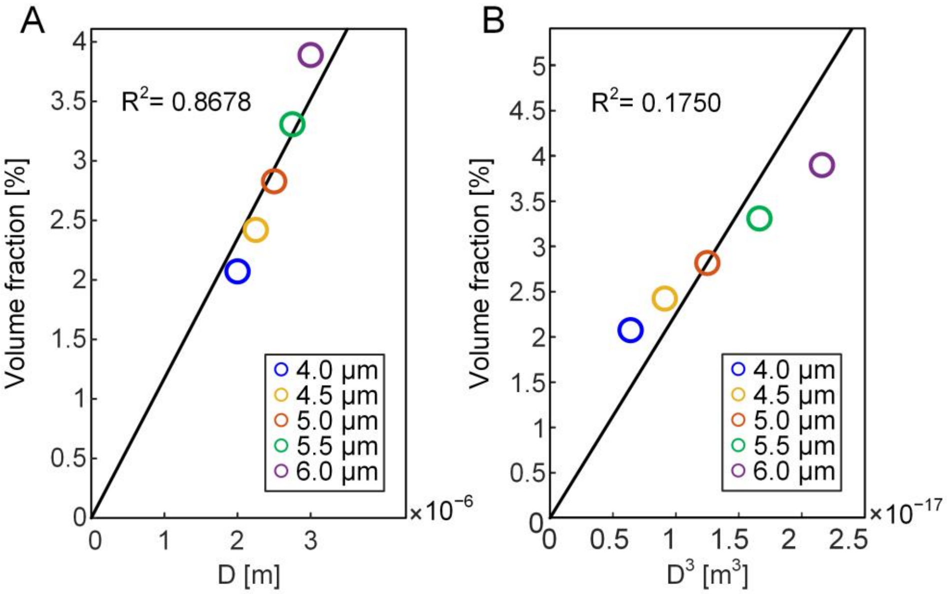

Supplementary Figure S4. Linear fitting of (A) cell diameter and (B) cell volume with the equivalent volume fraction.

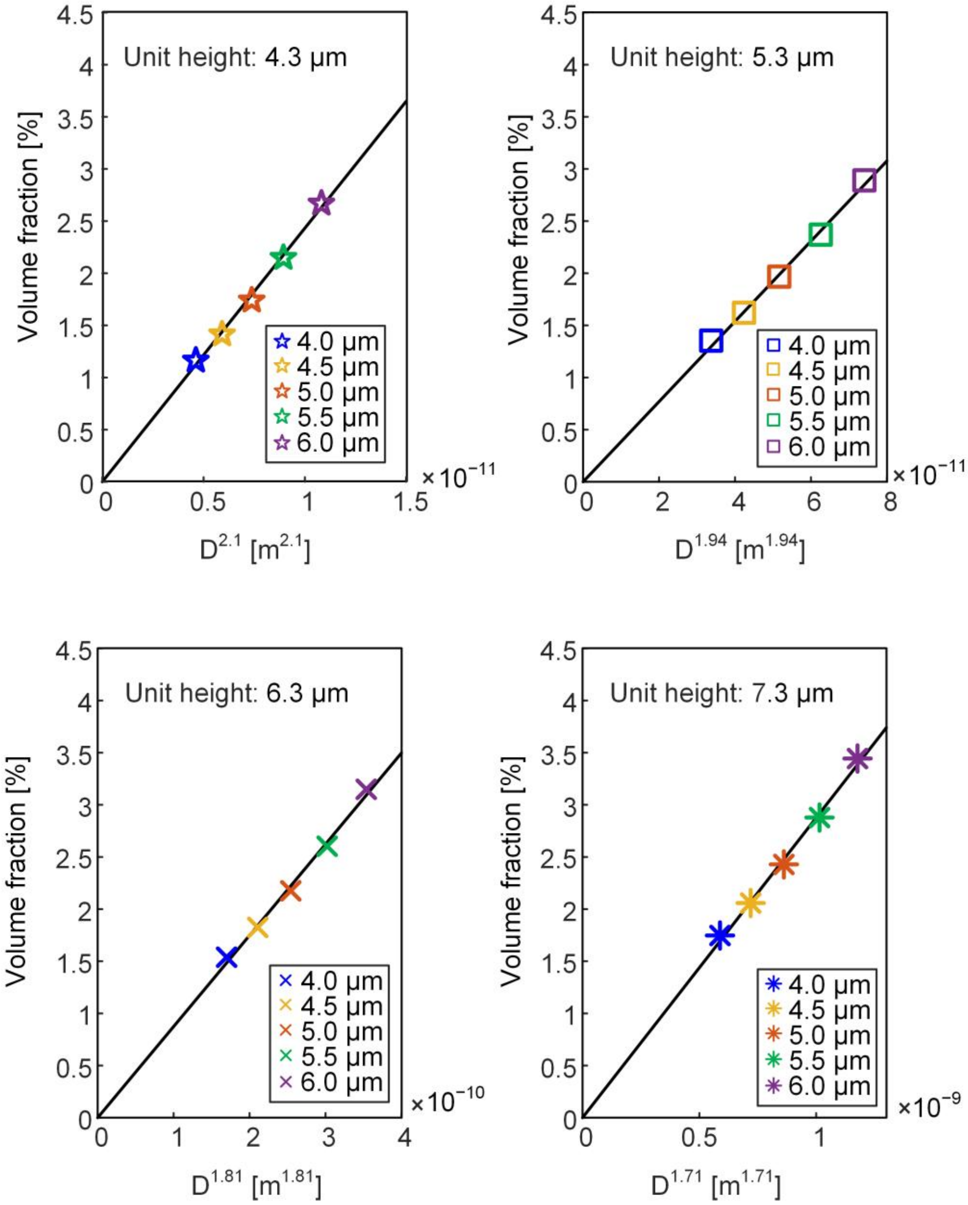

Supplementary Figure S5. Linear fitting of different geometric dimensions with the equivalent volume fraction across different trap heights (from 4.3 μm to 8.3 μm at a 1-μm interval).

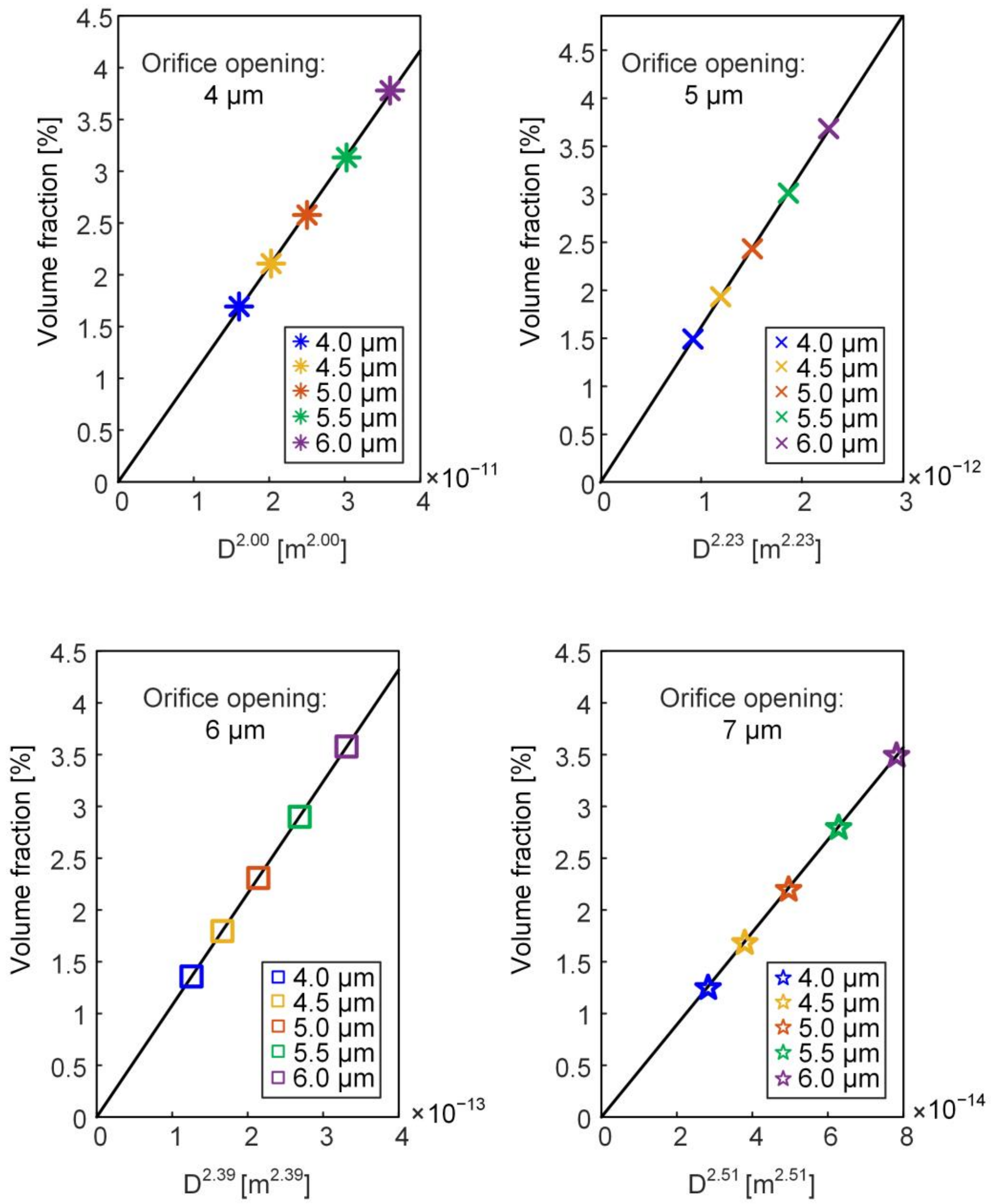

Supplementary Figure S6. Linear fitting of different geometric dimensions with the equivalent volume fraction across different orifice openings (from 3 μm to 7 μm at a 1-μm interval).
